# Paeoniflorin inhibits epithelial mesenchymal transformation and oxidative damage of lens epithelial cells in diabetic cataract via sirtuin 1 upregulation

**DOI:** 10.1080/21655979.2021.2018534

**Published:** 2022-02-19

**Authors:** Kun Zeng, Wenqun Xi, Yuanjiao Qiao, Xiaosheng Huang, Xinhua Liu

**Affiliations:** Cataract Department, Shenzhen Eye Hospital; Shenzhen Eye Institute; Shenzhen Eye Hospital Affiliated to Jinan University; School of Optometry, Shenzhen University, Shenzhen, Guangdong, China

**Keywords:** Paeoniflorin, diabetic cataract, lens epithelial cells, EMT, oxidative damage, SIRT1

## Abstract

Paeoniflorin (Pae) has been reported to serve an important role in complications associated with diabetes. To the best of our knowledge, the role of Pae in diabetic cataracts has not yet been reported. Human lens epithelial SRA01/04 cells were induced by high glucose (HG) and subsequently treated with Pae. Cell viability was detected using the MTT assay. Moreover, LDH levels were detected. Immunofluorescence (IF) and Western blotting were used to determine the protein expression levels of N-cadherin and E-cadherin. ELISA was performed to determine oxidative stress-related indicator levels. TUNEL and Western blotting detected the apoptotic rate. The mRNA and protein expression levels of sirtuin 1 (SIRT1) in SRA01/04 cells were measured via reverse transcription-quantitative PCR and Western blotting, respectively. Subsequently, cell transfection techniques were used to inhibit the expression of SIRT1 in cells. MTT, ELISA, IF, Western blotting and TUNEL assays were used to investigate the mechanisms of epithelial-mesenchymal transition (EMT) and oxidative damage with Pae in the diabetic cataract. Pae significantly increased cell viability and possibly inhibit the EMT and oxidative damage of SRA01/04 cells induced by HG. Pae was demonstrated to upregulate SIRT1 expression levels. The results therefore suggested that the downregulation of SIRT1 reversed the protective effect of Pae on EMT and oxidative damage in SRA01/04 cells induced by HG. In conclusion, Pae may inhibit EMT of lens epithelial cells and reduce oxidative damage in diabetic cataracts via the upregulation of SIRT1.

## Introduction

Diabetes mellitus refers to a group of metabolic diseases characterized by hyperglycemia. With improvements in the standard of living, as well as changes in lifestyle and diet structure, the incidence and mortality rate of diabetes is increasing annually [1]. Diabetes is associated with cardiovascular disease, diabetic nephropathy, diabetic cataracts, diabetic neuropathy and other complications. Cases of diabetes with such complications have higher morbidity and mortality rates than those cases without [2,3]. Diabetic cataract is one of the most important complications. The occurrence of this disease is closely related to the metabolism of the lens, and the clinical manifestations being similar to those seen in senile cataracts. The high incidence, rapid progression and ease of cataract maturation are the main characteristics of the disease and therefore early detection is vital [4]. Due to the high recurrence rate and the possibility of numerous postoperative complications, the task of developing therapeutic drugs for diabetic cataracts remains difficult.

Paeoniflorin (Pae) is the main active ingredient of *Radix paeoniae alba* and *Radix paeoniae rubra*. It functions as a sedative and an anti-inflammatory. Pae has been demonstrated to improve cognitive ability and protect the liver [5]. A previous study reported that Pae protects islet β cells from streptozocin-induced damage by inhibiting the p38 MAPK and JNK signaling pathways [6]. Pae serves a protective role in vascular endothelial injury induced by fluctuating hyperglycemia via antioxidant and anti-inflammatory activities, as well as via the reduction of protein kinase C β1 [7]. However, the role of Pae in diabetic cataracts has not been reported.

Compared with healthy mice, the expression levels of microRNA (miR)-211, Bax and p53 have been demonstrated to increase in diabetic cataract mice, whereas the expression levels of Bcl-2 and sirtuin 1 (SIRT1) decrease. miR-211 also can promote apoptosis and inhibit the proliferation of lens epithelial cells in diabetic cataract mice by targeting SIRT1 [8]. It can therefore be hypothesized that SIRT1 serves an important role in diabetic cataracts. Furthermore, numerous studies have reported that SIRT1 could potentially be a target for diabetic cataract therapeutics. *Lycium barbarum* polysaccharides have been demonstrated to prevent diabetic cataracts by upregulating SIRT1 and Bcl-2 and inhibiting genes related to cell death [9]. Blueberry anthocyanin extract mediated antioxidant activity by enhancing SIRT1 expression and decreasing NF-κB expression, which significantly delayed diabetic cataract progression [10]. Previous study has also demonstrated that Pae inhibited oxidized (ox)-low density lipoprotein (LDL)-induced apoptosis and the expression of adhesion molecules in HUVECs via SIRT1 upregulation [11]. It can therefore be hypothesized that Pae serves a role in diabetic cataracts by targeting SIRT1.

Thus, the aim of the present study was to determine the effect of Pae on cell transformation and oxidative damage by using a diabetic cataract cell model, inducing lens epithelial cells with high glucose (HG).

Materials and methods

*Cell culture*. The human lens epithelial SRA01/04 cell line (BeNa Culture Collection; Beijing Beina Chunglian Institute of Biotechnology) was cultured in DMEM supplemented with 10% FBS in a humidified, 37°C, 5% CO_2_ incubator. SRA01/04 cells was cultured in medium containing 5.5 (control) and 30 mM glucose (HG group) and were then treated with different concentrations of Pae (0.1, 1 and 10 µM) for 24 h or N-acetyl cysteine (NAC; 1 mM) for 24 h. Cells were also treated with mannitol (MA group) at the same concentration as high glucose group.

*MTT assay*. The cytotoxic effects of Pae were evaluated using the MTT assay [12]. SRA01/04 cells were seeded into 96-well plates at a density of 1 × 10^5^ cells/well. Following treatment with HG and Pae for 24 h, 10 μl of MTT solution (5 mg/ml) was added to each well and the cells were incubated for 4 h. Subsequently, DMSO (150 μl) was added following removal of the supernatant. Absorbance was measured at 490 nm with a microplate analyzer (Thermo Fisher Scientific, Inc.).

*Lactate dehydrogenase (LDH) assay*. A CytoTox 96 Non-Radioactive Cytotoxicity Assay kit (cat. no. G1780; Promega Corporation) was used to detect LDH levels according to the manufacturer’s protocol [13]. Cells were seeded into 96-well plates at a density of 1 × 10^3^ cells/well and pre-treated with Pae before HG induction. Subsequently, 10 µl cell supernatant was mixed with 100 µl LDH reaction reagent at 25°C for 30 min. The absorbance was determined using an ELISA reader (Victor X3; PerkinElmer, Inc.) with a 490 nm filter.

*Immunofluorescence (IF) staining*. SRA01/04 cells were fixed using 4% paraformaldehyde for 30 min and subsequently permeabilized with PBS containing 0.1% Triton X-100. Cells were then blocked with 2% BSA (Beyotime Institute of Biotechnology) at 25°C. Subsequently, cells were further incubated with anti-E-cadherin antibody (cat. no. ab40772; Abcam) and anti-N-cadherin (cat. no. ab18203; Abcam) antibody overnight at 4°C. Following the primary incubation, cells were incubated with secondary antibody (cat. no. ab97174; Abcam) for 1 h at room temperature. Nuclei were visualized using DAPI (Sigma-Aldrich; Merck KGaA). Images were obtained using a Laser Scanning Confocal Microscope (Leica Microsystems GmbH) [14].

*Western blotting*. SRA01/04 cells were lysed with RIPA buffer (Beyotime Institute of Biotechnology) with a protease inhibitor cocktail (Sigma-Aldrich; Merck KGaA). The BCA Protein Assay kit (ProteinTech Group, Inc.) was used to quantify total protein. Total protein (30 μl) was separated using SDS‐PAGE on a 10% gel. Separated proteins were subsequently transferred to a PVDF membrane and blocked with skimmed milk powder for 1.5 h. The membranes were incubated with the following primary antibodies against: E-cadherin (1:1,000; Origene Technologies, Inc.), N-cadherin (1:1,000; Cell Signaling Technology, Inc.), Vimentin (1:1,000; Cell Signaling Technology, Inc.), Snail (1:1,000; Abcam), Bax (1:1,000; Abcam), cleaved (c)-caspase 9 (1:1,000; Abcam), cytochrome *c* (1:1,000; Abcam), caspase 9 (1:1,000; Abcam), Bcl2 (1:1,000; Abcam) and SIRT1 (1:1,000; Abcam) overnight at 4°C. Protein bands were visualized using the ECL kit (Santa Cruz Biotechnology, Inc.) and Image Laboratory analysis software (Bio‐Rad Laboratories, Inc.) following 1.5 h incubation with the secondary antibody (1:5,000; Abcam).

*Oxidative stress index detection*. Glutathione peroxidase (GSH-px; cat. no. S0073), malondialdehyde (MDA; cat. no. S0131M) and superoxide dismutase (SOD; cat. no. S0086) levels in SRA01/04 cells were quantified using commercial kits purchased from the Beyotime Institute of Biotechnology according to the manufacturer’s protocol.

*TUNEL assay*. Apoptotic SRA01/04 cells were detected using the TUNEL assay with the Fluorometric TUNEL System (Promega Corporation) according to the manufacturer’s protocol [15]. Briefly, 50 µl recombinant terminal deoxynucleotidyl transferase incubation buffer were added to the permeabilized cells on the slides, which were then incubated under a humidified atmosphere for 60 min in the dark and subsequently rinsed three times with 2X saline-sodium citrate buffer. The slides were subsequently stained with DAPI and imaged under a fluorescence microscope (BX51; Olympus Corporation). TUNEL-positive cells were indicated by the emission of green fluorescence and the nuclei were indicated by blue fluorescence.

*Reverse transcription-quantitative PCR (RT-qPCR)*. Total RNA was extracted from SRA01/04 cells using TRIzol® reagent (Invitrogen; Thermo Fisher Scientific, Inc.) according to the manufacturer’s protocol. Total RNA was reverse transcribed into cDNA using the M-MLV First Chain Synthesis kit (Invitrogen; Thermo Fisher Scientific, Inc.). qPCR was performed with the StepOnePlus Real-Time PCR System (Applied Biosystems; Thermo Fisher Scientific, Inc.) and iTaq SYBR Green Supermix (Applied Biosystems; Thermo Fisher Scientific, Inc.). The thermocycling conditions were used for qPCR as follows: 95°C for 2 min; 40 cycles at 95°C for 20 sec, 60°C for 15 sec and 72°C for 30 sec. Primers were synthesized by Sangon Biotech Co., Ltd. Primer sequences were as follows: SIRT1 forward (F), 5ʹ-CCTTTCAGAACCACCAAAGCGGAA-3ʹ and reverse (R), 5ʹ-AGTCAGGTATCCCACAGGAAACAG-3ʹ; and GAPDH F, 5ʹ-AGGTCGGTGTGAACGGATTTG-3ʹ and R, 5ʹ-TGTAGACCATGTAGTTGAGGTCA-3ʹ. GAPDH was used as internal reference gene to normalize gene expression [16].

*Cell transfection*. The short hairpin RNA (shRNA) expression plasmids, shRNA-SIRT1#1 and shRNA-SIRT1#2, were obtained from Guangzhou RiboBio Co., Ltd. shRNA-SIRT1#1, shRNA-SIRT1#2 and the shRNA-negative control (NC) were transfected into SRA01/04 cells at a final concentration of 50 nM for 48 h by using Lipofectamine® 2000 according to the manufacturer’s protocol. RT-qPCR and Western blotting detected the effect of the transfection.

*Statistical analysis*. SPSS version 21.0 (IBM Corp.) was used to perform all statistical analyses. Data are presented as the mean ± SD. For statistical comparisons between ≥3 groups, one-way ANOVA followed by Tukey’s post hoc test was used. P < 0.05 was considered to indicate a statistically significant difference. Each experiment was repeated three times.

## Results

**Figure 1.**
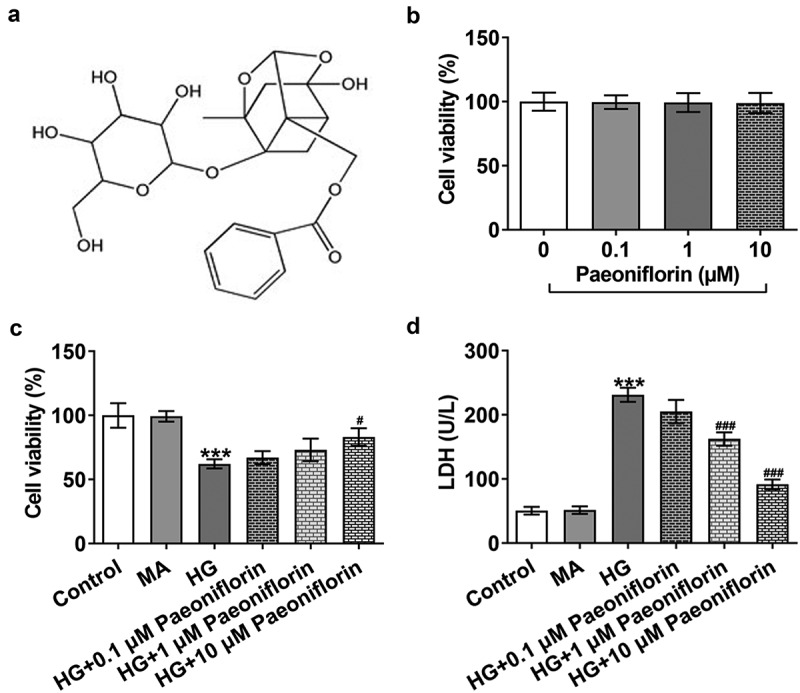
Pae increased the activity of SRA01/04 cells induced by HG. a. The chemical formula for Pae. b. MTT detected the viability of cells treated by Pae. c. MTT detected the viability of HG induced cells treated by Pae. d. The release of LDH was detected by kit. ***p < 0.001 vs MA, #p < 0.05, ###p < 0.001 vs HG.

*Pae increases the activity of SRA01/04 cells induced by HG*. The chemical formula of Pae is displayed in Figure 1(a). Cells were induced with different concentrations of Pae. The results of the MTT assay demonstrated that Pae had no significant effect on cell viability (Figure 1(b)). The effect of Pae on HG-induced cells was subsequently examined. The results demonstrated that the viability of the HG group was significantly decreased compared with the MA group. Compared with the HG group, the cell viability increased significantly in a Pae dose-dependent manner (Figure 1(c)). Moreover, LDH levels were detected. The results demonstrated that compared with the HG group LDH levels were obviously increased in the MA group. Compared with the HG group, LDH levels gradually decreased following Pae administration (Figure 1(d)). As 10 µM Pae had the most significant effect in these aforementioned experiments, 10 µM Pae treated for 24 h was selected for subsequent experimentation.

**Figure 2.**
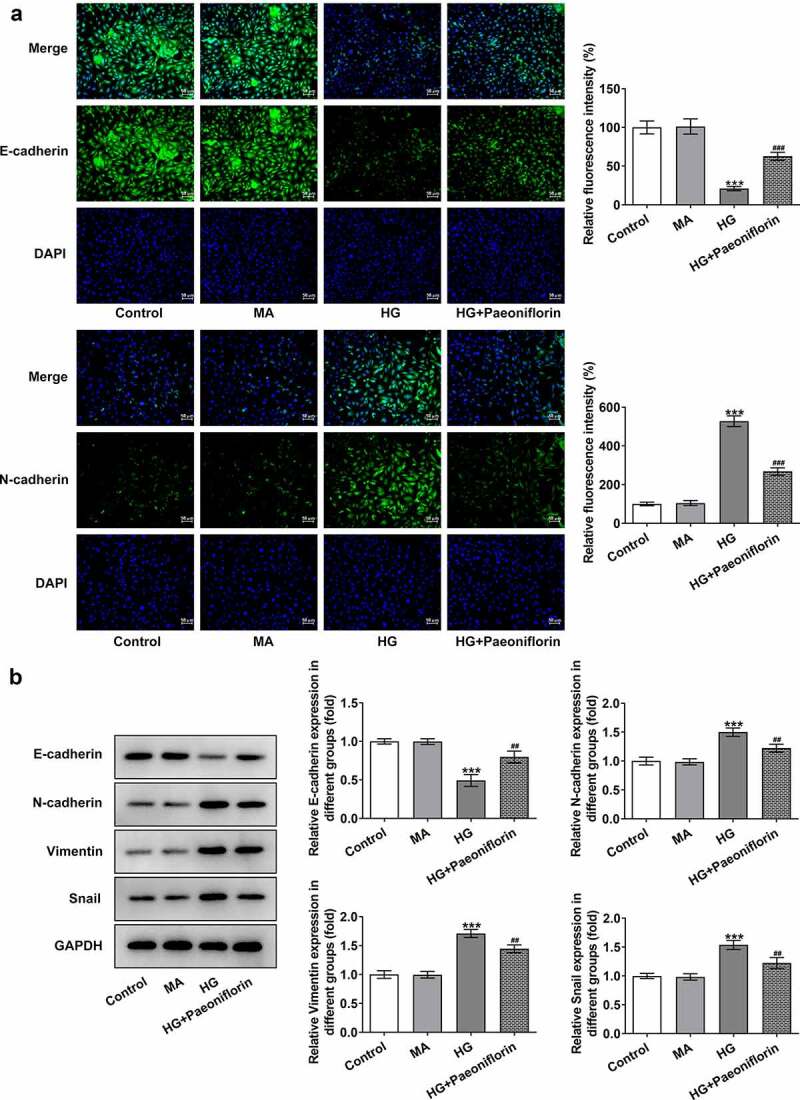
Pae inhibited EMT in SRA01/04 cells induced by HG. a. IF detected the expression of E-cadherin and N-cadherin. b. Western blot detected the expression of EMT-related proteins. ***p < 0.001 vs MA, ##p < 0.01, ###p < 0.001 vs HG.

*Pae inhibits the epithelial-mesenchymal transition (EMT) in SRA01/04 cells induced by HG*. To examine the effect of Pae on the EMT, IF was used to detect the expression of EMT-related proteins E-cadherin and N-cadherin. The results demonstrated that the protein expression levels of E-cadherin decreased and the protein expression levels of N-cadherin increased following HG induction compared with the MA group. Compared with the HG group, E-cadherin protein expression levels were increased and N-cadherin protein expression levels were decreased in the HG + Pae group (Figure 2(a)). These results are consistent with those determined by the Western blotting analysis. Furthermore, compared with the MA group, the expression of Vimentin and Snail increased following HG induction. The expression of Vimentin and Snail in the HG + Pae group decreased compared with the HG group (Figure 2(b)).

**Figure 3.**
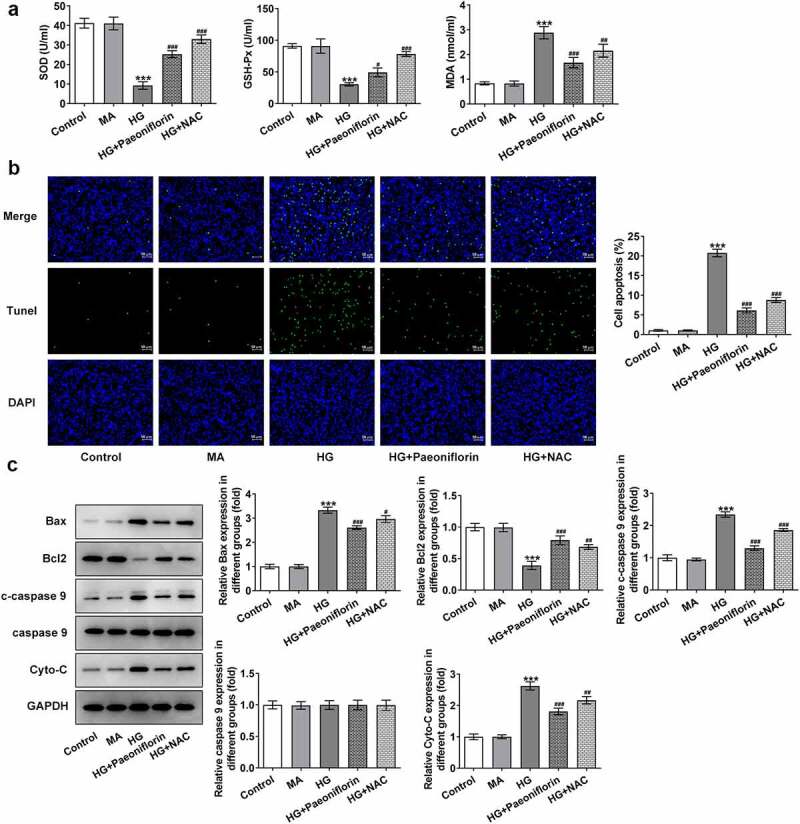
Pae inhibited oxidative damage in SRA01/04 cells induced by HG. a. ELISA kits detected the expression of SOD,GSH-Px and MDA. b. Tunel assay detected the apoptosis of cells. c. Western blot detected the expression of apoptosis-related proteins. ***p < 0.001 vs MA, #p < 0.05, ##p < 0.01, ###p < 0.001 vs HG.

*Pae inhibits oxidative damage in SRA01/04 cells induced by HG*. SOD, GSH-Px and MDA levels were detected using ELISA to explore the effect of Pae on cell oxidative stress injury. The results demonstrated that compared with the MA group, the levels of SOD and GSH-Px in the HG group were significantly decreased, whereas MDA levels were increased. However, compared with the HG group, the effects on the SOD, GSH-Px and MDA levels in the HG + Pae and HG + NAC groups were reversed following treatment (Figure 3(a)). TUNEL and Western blotting results demonstrated that compared with the MA group, apoptosis was significantly increased in the HG group, accompanied by an increase in the protein expression levels of Bax, c-caspase 9 and cytochrome *c* and a decrease in Bcl-2 protein expression levels. Compared with the HG group, the effect of apoptosis was significantly reversed in the HG + Pae and HG + NAC groups following treatment (Figure 3(b,c)). These results indicated that Pae may inhibit oxidative damage induced by HG in SRA01/04 cells.

**Figure 4.**
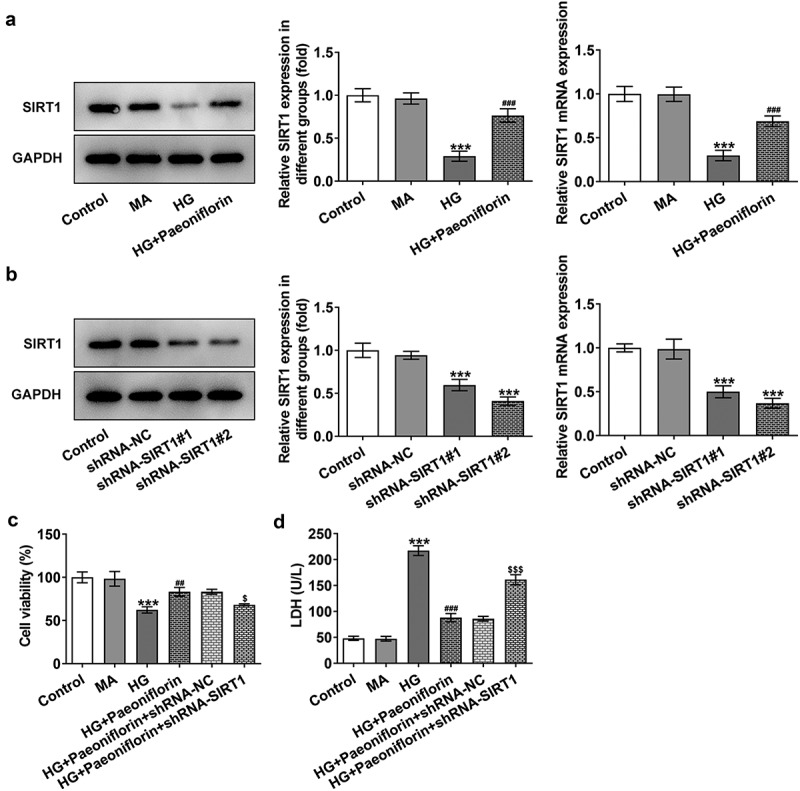
Down-regulation of SIRT1 reversed the protective effect of Pae on the activity in SRA01/04 cells induced by HG. a. Western blot and RT-qPCR detected the expression of SIRT1 of HG inducted cells treated by Pae. ***p < 0.001 vs MA, ###p < 0.001 vs HG. b. Western blot and RT-qPCR detected the expression of SIRT1 after transfection. ***p < 0.001 vs shRNA-NC. c. MTT detected the viability of cells. d. The release of LDH was detected by kit. ***p < 0.001, ##p < 0.01, ###p < 0.001vs HG, $p < 0.05, $$$p < 0.001 vs HG + Paeoniflorin + shRNA-NC.

**Figure 5.**
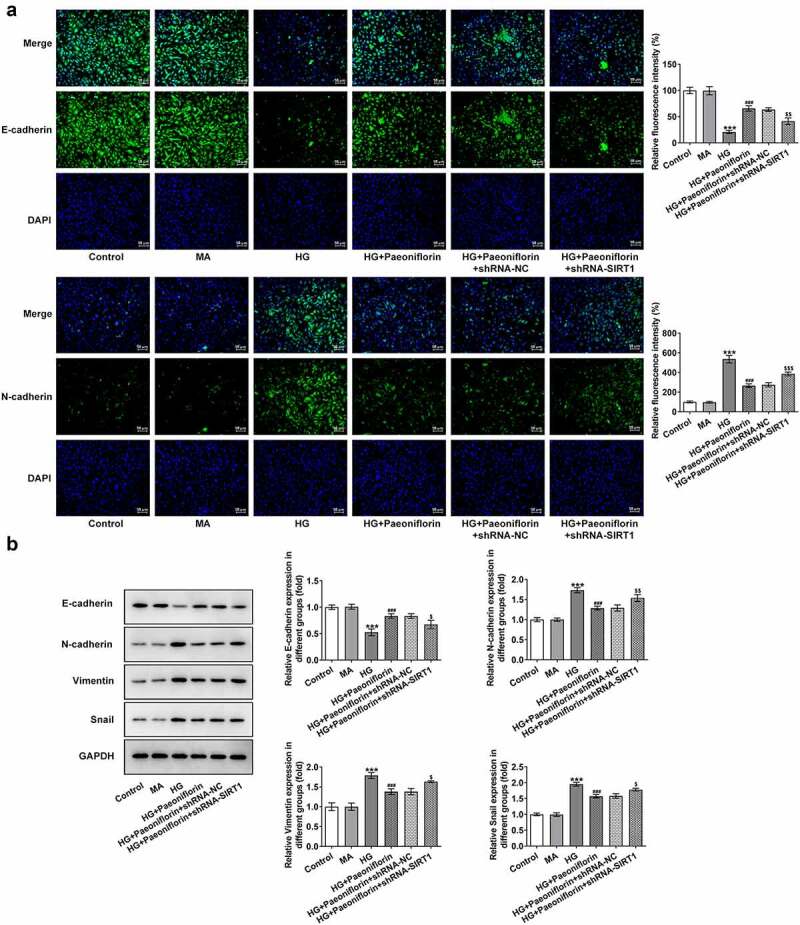
Down-regulation of SIRT1 reversed the protective effect of Pae on EMT in SRA01/04 cells induced by HG. a. IF detected the expression of E-cadherin and N-cadherin. b. Western blot detected the expression of EMT-related proteins. ***p < 0.001, ##p < 0.01, ###p < 0.001vs HG, $p < 0.05, $$p < 0.01, $$$p < 0.001 vs HG + Paeoniflorin + shRNA-NC.

**Figure 6.**
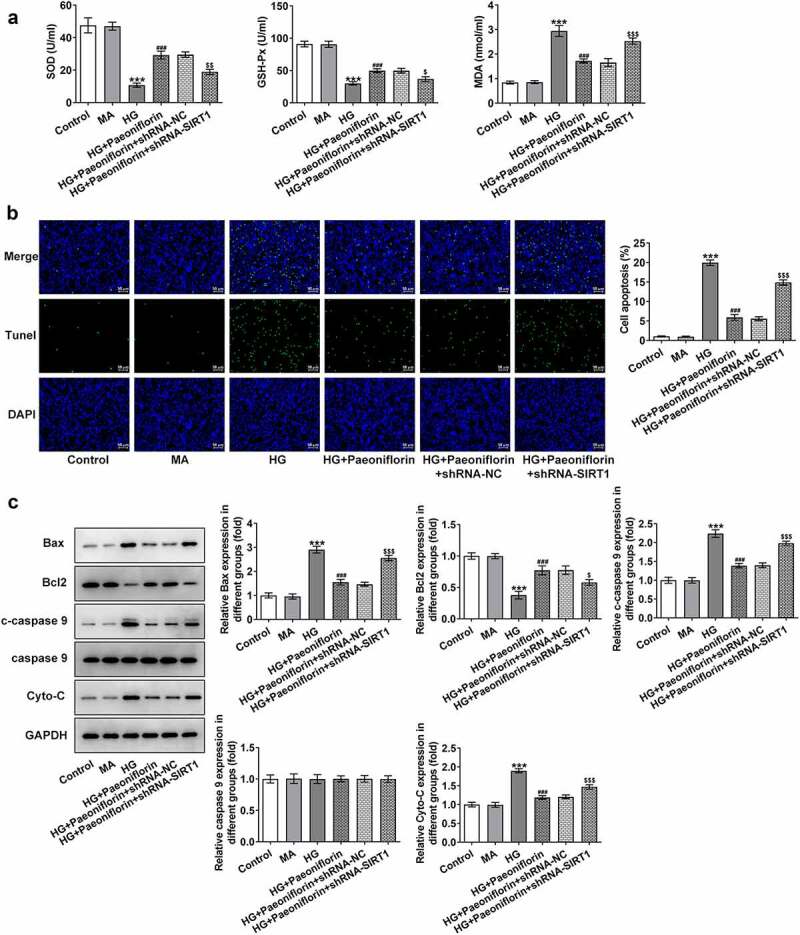
Down-regulation of SIRT1 reversed the protective effect of Pae on oxidative damage in SRA01/04 cells induced by HG. a. ELISA kits detected the expression of SOD,GSH-Px and MDA. b. Tunel assay detected the apoptosis of cells. c. Western blot detected the expression of apoptosis-related proteins. ***p < 0.001, ##p < 0.01, ###p < 0.001vs HG, $p < 0.05, $$p < 0.01, $$$p < 0.001 vs HG + Paeoniflorin + shRNA-NC.

*Knockdown of SIRT1 reverses the protective effect of Pae on the EMT and oxidative damage in SRA01/04 cells induced by HG*. Throughout the experiment, SIRT1 expression was determined to be abnormal. Compared with the MA group, SIRT1 expression levels in the HG group were significantly decreased. Compared with the HG group, SIRT1 expression levels in the HG + Pae group were significantly increased (Figure 4(a)). To further explore the mechanism of Pae in diabetic cataract, cell transfection was used to knockdown SIRT1 mRNA expression in cells (Figure 4(b)). Compared with shRNA-NC, SIRT1 expression levels in the shRNA-SIRT1#1 and shRNA-SIRT1#2 groups were significantly decreased, with the decrease in shRNA-SIRT1#2 group being greater. shRNA-SIRT1#2 was therefore selected for subsequent experiments. Moreover, the results demonstrated that compared with the HG + Pae + shRNA-NC group, cell viability in the HG + Pae + shRNA-SIRT1 group was decreased (Figure 4(c)) and LDH levels were increased (Figure 4(d)). Subsequently, EMT-related proteins were detected and the results demonstrated that the expression of E-cadherin in the HG + Pae + shRNA-NC group was significantly decreased compared with the HG + Pae + shRNA-SIRT1 group. The expression of N-cadherin, Vimentin and Snail was increased significantly (Figure 5(a,b)). Oxidative stress assays demonstrated that SOD and GSH-Px levels were decreased, whereas MDA levels were increased in the HG + Pae + shRNA-SIRT1 group compared with the HG + Pae + shRNA-NC group (Figure 6(a)). TUNEL and Western blotting analyses demonstrated that, compared with the HG + Pae + shRNA-NC group, apoptosis and Bax, c-caspase 9 and cytochrome *c* protein expression levels were increased, whereas Bcl2 protein expression levels were decreased in the HG + Pae + shRNA-SIRT1 group (Figure 6(b,c)).

## Discussion

Lens epithelial cells are the most active component in lens metabolism. They can produce lens fibers, maintain the metabolism of the whole lens and serve an important role in maintaining lens transparency [17,18]. A previous study reported that the oxidative stress injury of lens epithelial cells caused by a long-term HG environment is the main cause of lens opacity [19]. Continuous hyperglycemia can accelerate the development of cataracts by altering the lens osmotic pressure and inducing lens oxidative stress [20,21]. In the present study the lens epithelial SRA01/04 cell line was induced by HG, which significantly decreased cell viability and resulted in oxidative damage to the cells. A close relationship between cataracts and the EMT has previously been determined [22]. Compared with normal lenses, diabetic lenses are more susceptible to oxidative stress, which is caused by antioxidant dysfunction [23]. The results of the present study demonstrated that HG-induced SRA01/04 cells underwent the EMT and the expression levels of EMT-related proteins increased.

Pae has anti-inflammatory and antioxidant properties and has been widely used in the treatment of diabetic complications. Pae can improve the infiltration and activation of macrophages in diabetic nephropathy through inhibiting the Toll-like receptor 4 (TLR4) signaling pathway [24]. Pae inhibits MMP-9 expression and the inflammatory response in hyperglycemia-induced retinal microglia via inhibition of the TLR4/NF-κB signaling pathway, through the upregulation of suppressor of cytokine signaling 3 in diabetic retinopathy [25]. Moreover, Pae alleviates oxidative stress, mitochondrial dysfunction and endoplasmic reticulum stress in retinal pigment epithelial cells by triggering Ca^2+^/calmodulin-dependent protein kinase II dependent AMPK activation [26]. To the best of our knowledge, the effect of Pae on lens epithelial cells induced by HG has not been reported. In the present study it was demonstrated that Pae could significantly inhibit cell activity, improve oxidative damage and inhibit the EMT of SRA01/04 cells induced by HG. These results indicated that Pae may have a therapeutic effect on diabetic cataracts.

In order to further investigate the mechanism of Pae on lens epithelial cells induced by HG, SIRT1 expression was explored as it was demonstrated to be abnormally expressed during the experiment. SIRT1 expression levels in cells induced by HG were obviously increased, which was also consistent with the results of Han *et al* [27]. SIRT1 serves an important role in diabetic cataracts and can be used as a therapeutic target. Blueberry anthocyanin extract mediates antioxidant activity by enhancing SOD and GSH activities, SIRT1 expression levels and decreasing NF-κB expression levels, which can significantly delay the progression of diabetic cataracts [28]. Compared with healthy mice, SIRT1 expression levels are decreased in diabetic cataract model mice. Targeting SIRT1 can promote the apoptosis and inhibit the proliferation of lens epithelial cells in these diabetic cataract model mice [29]. Furthermore, a previous study has demonstrated that Pae inhibits ox-LDL-induced apoptosis and the expression of adhesion molecules of Human umbilical vein endothelial cells via the upregulation of SIRT1 [30]. In the present study, the results demonstrated that the downregulation of SIRT1 could reverse the protective effect of Pae on the EMT and oxidative damage of SRA01/04 cells induced by HG.

Study has shown that high glucose can induce ETM of lens epithelial cells, leading to the fibrosis and turbidity of lens, which is an important mechanism of diabetic cataract [31]. Therefore, EMT expression of HG-induced SRA01/04 cells was also detected in our paper. We found that Pae inhibited the expression of E-cadherin and promoted the expression of N-cadherin, thus inhibiting the EMT of SRA01/04 cells induced by HG. In addition, study have shown that SIRT1 can accelerate the autophagic degradation of E-cadherin by deacetylating Beclin1 [32]. The paradox is that SIRT1 can be used as the promoter of E-cadherin in endometrial cancer cells to activate its expression [33]. The results of our paper showed that inhibition of SIRT1 could significantly reverse the activation effect of Pae on E-cadherin expression, and N-cadherin expression was significantly increased at this moment. These results suggested that Pae might inhibit HG-induced EMT of SRA01/04 cells by regulating SIRT1 expression.

This article also has some limitations. We did not demonstrate our conclusions in animals or in the lens of diabetic cataract patients. And this will be discussed in future experiments. Moreover, we will further explore the regulatory relationship between SIRT1 and cadherin in the process of EMT in future experiments.

## Conclusion

In conclusion, the present study indicated that Pae may inhibit EMT and reduce oxidative damage of lens epithelial cells in diabetic cataracts via the upregulation of SIRT1. Pae may therefore be a potential therapeutic for the treatment of diabetic cataracts.

## Data Availability

The datasets analyzed during the current study are available from the corresponding author on reasonable request.

## Highlights

Paeoniflorin inhibits epithelial mesenchymal transformation in diabetic cataract

Paeoniflorin inhibits oxidative damage in diabetic cataract

Paeoniflorin upregulates the expression of SIRT1
